# Successful Treatment of Adult-Onset Recurrent Respiratory Papillomatosis with CO_2_ Laser and Photodynamic Therapy

**DOI:** 10.1155/2019/7394879

**Published:** 2019-10-16

**Authors:** Sheng Lu, Yang Liu, Runjie Shi, Pingyu Zhou

**Affiliations:** ^1^Department of Sexually Transmitted Disease, Tongji University Affiliated Shanghai Skin Disease Hospital, Shanghai 200443, China; ^2^Department of Dermatology, Shanghai Ninth People's Hospital Affiliated to Shanghai Jiaotong University School of Medicine, Shanghai 200011, China; ^3^Department of Otolaryngology, Shanghai Ninth People's Hospital Affiliated to Shanghai Jiaotong University School of Medicine, Shanghai 200011, China

## Abstract

Recurrent respiratory papillomatosis is a noninvasive benign epithelial tumor caused by human papillomavirus. Clinically, it featured rapid growth, multifocus, and frequent recurrence. Though a number of therapies have been investigated, the recurrence after treatment is always a challenge. In this report, we describe a 27-year-old male patient with recurrent respiratory papillomatosis who was treated with CO_2_ laser therapy followed by 5-aminolevulinic acid photodynamic therapy (ALA-PDT). There was no adverse reaction after treatment and no recurrence during the follow-up time.

## 1. Introduction

Caused by human papillomavirus (HPV), recurrent respiratory papillomatosis (RRP) is a benign and rare disease, which is characterized by exophytic epithelial lesions affecting the larynx and may lead to hoarseness, coughing, wheezing, voice change, chronic dyspnoea, choking, syncope, and many other disorders [1]. Though HPV-6 and HPV-11 are the most often detected virus subtypes in about 90% of the cases [2], RRP does have a malignant transformation manner. According to the age at onset, RRP can be divided into juvenile and adult types [3]. The incidence of RRP has been estimated to range from 1 to 4 per 100,000 among children [4] and 1.8 cases per 100,000 among adults [5].

Traditional managements of RRP are just simply removal of the lesions, including cryotherapy, laser microsurgery, or surgery with a microdebrider. [6, 7]. However, RRP tends to reoccur in most of the patients after above treatments [8], which is always a challenge clinically. Photodynamic therapy (PDT) is a noninvasive treatment for a wide variety of malignancies and premalignant dysplasia [9]. The PDT combined with CO_2_ laser therapy was approved to be a very effective way in stopping the recurrence of genital warts [9–11]. In this report, we present a case of RRP in a male patient, who underwent surgical excision twice in about 4 months and was then treated with CO_2_ laser and PDT. After CO_2_ laser and PDT treatment, the patients showed no recurrence during the 15-month follow-up.

## 2. Case Report

**Figure 1:**
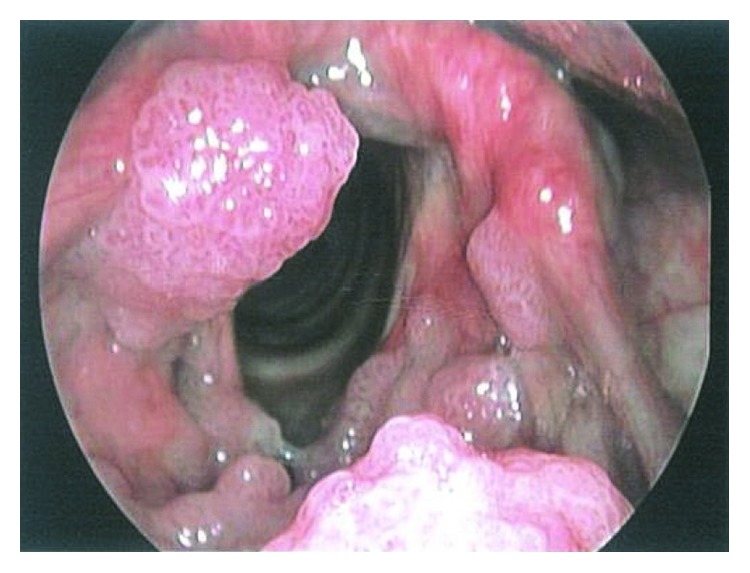
The appearance of lesion the first time the patient visited the hospital; warts located at the right ventricular band, inside of the arytenoid cartilage, and on the laryngeal side of the epiglottis.

**Figure 2:**
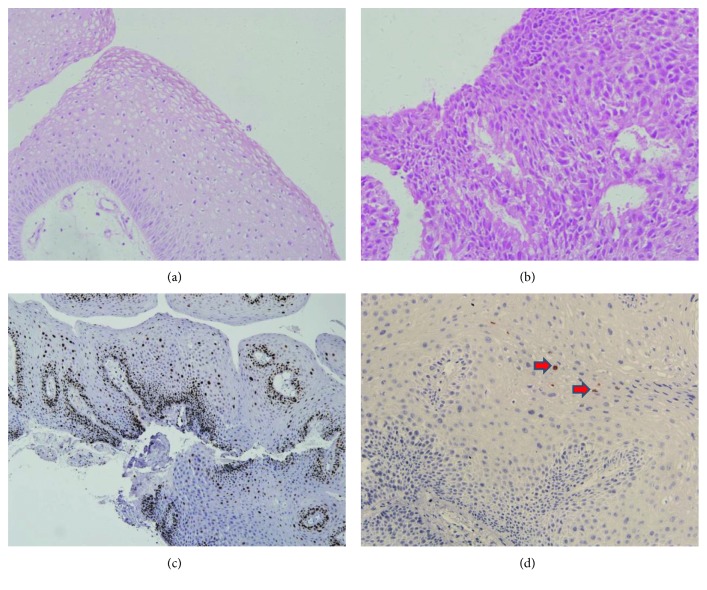
Initial biopsy during the first visit (a) Vacuolar degeneration in epithelium cells (magnification ×200). (b) Mild atypical hyperplasia in epithelium (magnification ×400). (c) Ki-67 antibody labelling index was 10% (magnification ×100). (d) Human papillomavirus (HPV) was detected in the patient's laryngeal tissue sample (red arrow) (magnification ×200).

In May 2017, a 27-year-old male presented with a 2-week history of progressive throat irritation and hoarseness. He stated that he first noticed foreign body sensation in the throat after a cold and then the discomfort became progressive and affected his voice. The patient then went to a local hospital and a direct laryngoscope examination was performed, which showed that inside of the arytenoid cartilage, on the laryngeal side of the epiglottis, and at the right ventricular band were located multiple masses of warty lesions with a cauliflower-like appearance (Figure 1). The patient was diagnosed with RRP, and neoplasm excision was performed. Histopathological examination was performed, and the pathology report showed papillomatous hyperplasia of the mucous epithelium, hypergranulosis, mild atypical hyperplasia (Figure 2(b)), and vacuolar degeneration in epithelium cells were observed (Figure 2(a)). Further immunohistochemical staining showed the Ki-67 antibody labelling index was 10% (Figure 2(c)), and human papillomavirus (HPV) was detected and was HPV-16-positive (Figure 2(d)). After surgery, the patient was failed to be followed up until he suffered hoarseness again and difficulty swallowing for four months. Then, he went to the previous hospital again and was given the same treatment. However, two months after the treatment, the above symptoms reoccurred and even worsened.

**Figure 3:**
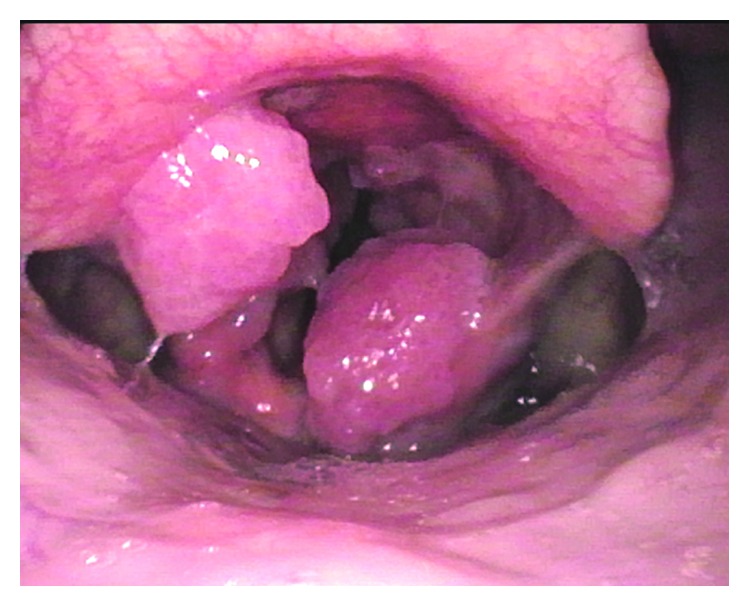
The appearance of recurrent lesion when the patient visited our hospital; the lesion was located at the laryngeal side of the epiglottis and inside of the arytenoid cartilage.

**Figure 4:**
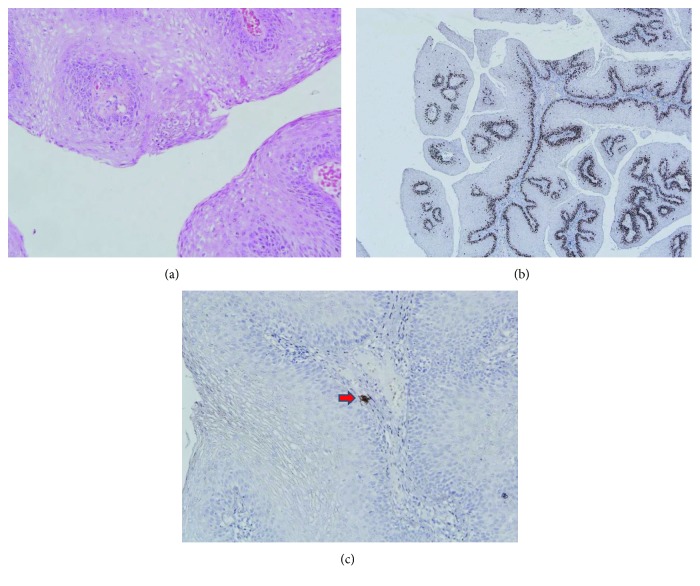
Biopsy of the patient when the patient visited our hospital. (a) Magnification ×200 showing the similar appearance like the fist time. (b) Magnification ×40 showing Ki-67 is 10% positive in the sample. (c) Magnification ×200 showing HPV-16 was found in the lesion (red arrow).

In January 2018, the patient visited our hospital. On inquiry, the patient denied any significant past medical history including diseases associated to RRP such as asthma and reflux esophagitis. He also denied fevers, rashes, and headache during the course of the disease. Though he preferred hot and spicy food, he denied any other exacerbating or alleviating factors. He had oral sex with his female sex partner who had genital warts at the same time. The patient denied a history of smoking or alcohol abuse. On examination, he was negative in the rapid plasma reagin test (RPR), Treponema pallidum particle agglutination assay (TPPA), and human immunodeficiency virus (HIV) antibody test. Repeat laryngoscopy showed the neoplasm resurfaced and the lesion located at the laryngeal side of the epiglottis and inside of the arytenoid cartilage (Figure 3). The lesions were taken using a laryngoscope for biopsy. The pathological changes showed papillomatous hyperplasia of the mucous epithelium and vacuolar degeneration in epithelium cells as noted the first time (Figure 4(a)). The histopathological change showed the characteristics of HPV infection, the immunohistochemical findings showed the lesion with a low proliferation index (Ki-67 10% positive) (Figure 4(b)), and HPV-16 was found in the lesion (Figure 4(c)). We also detected HPV-DNA in the neoplasm sample with polymerase chain reaction (PCR) technique, and HPV-16 subtype was also found. So, the patient was diagnosed as recurrent respiratory papillomatosis.

**Figure 5:**
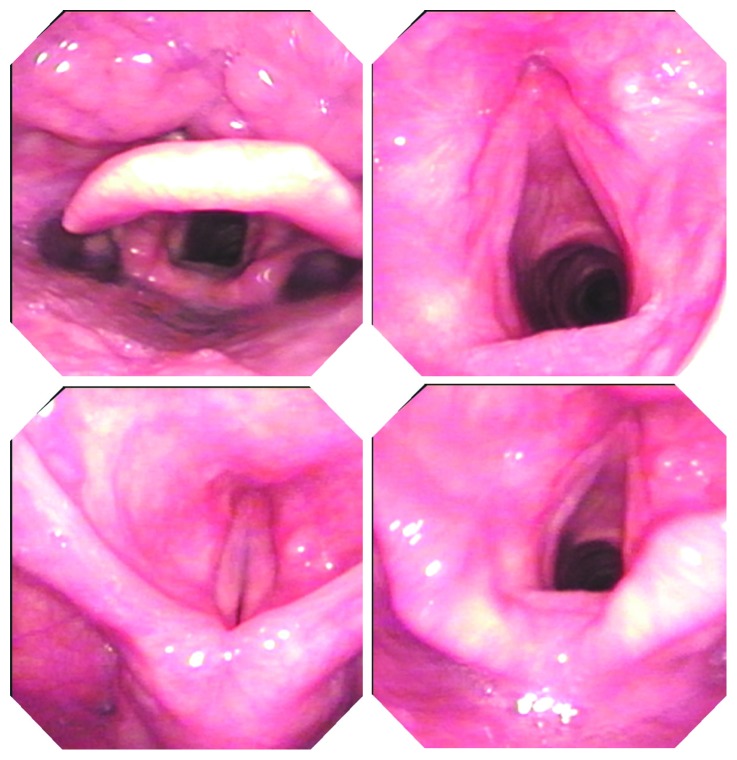
Six months after therapy. No recurrence was found in the epiglottis and around the glottis.

As the patient experienced multiple recurrences, CO_2_ laser therapy followed by PDT was given to prevent the recurrence of the neoplasm. After the neoplasm was removed by CO_2_ laser under general anesthesia, self-retaining laryngoscope PDT was performed at the surgical site. The detailed procedure is as follows: aminolevulinic acid (ALA) hydrochloride powder (118 mg; Shanghai Fudan Zhangjiang Bio-Pharmaceutical Co., Ltd.) was dissolved into sterile water for a final solution of 20% concentration and then applicated on to the operative region and surrounding mucosa by a piece of sterilized ribbon gauze for three hours. At last, the lesion received an irradiation of 635 nm wavelength and 120 J/cm2 power for 30 minutes. There were no adverse reactions and complications after PDT. After the treatment, the patient was asked to make a follow-up visit every two weeks for six months. There was no recurrence found under laryngoscopy after six months of the surgery (Figure 5), and recurrence was not found during telephone follow-up 15 months after CO_2_ laser therapy and PDT treatment.

## 3. Discussion

The viral etiology of RRP was first described by Ullmann in 1923 [12]; electron microscopy, in situ hybridization, and polymerase chain reaction techniques confirmed the hypothesized role of HPV later [6]. HPV can be divided into high-risk and low-risk based on its ability to cause malignant transformation of epithelial cells. Though most adult-onset laryngeal papillomatosis cases were benign [13], the high-risk HPV types 16, 18, 31, and 33 have been reported in RRP [14], and they may cause potential malignant transformation in less than 1% of cases [14]. The risk factors of malignant transformation in RRP are believed to be (1) radiation; (2) immunosuppression; (3) chemotherapy; (4) multiple recurrences; and (5) smoking and/or alcohol addiction [15]. Though the pathological findings of our case showed noninvasive benign changes, immunohistochemical examination reported the proliferation index (Ki-67) at a low level; HPV-16 and atypical hyperplasia were identified in the lesion, and the patient had multiple recurrences. Therefore, photodynamic therapy was provided for the patient after CO_2_ laser was used, for PDT is believed to not only prevent the recurrence but also the malignant transformation.

PDT is a minimally invasive treatment modality that depends on the interaction of a nonthermal laser light of a specific wavelength with a light-activated photosensitizer. The excitation of the photosensitizer causes the production of cytotoxic reactive oxygen species (ROS), such as singlet molecular oxygen, hydroxyl radicals, or superoxide anions, which achieve photocytotoxicity through oxidatively stressing target cells and induce damage to their cellular biomolecules, thus achieving a therapeutic effect [16]. Many researchers have proved that PDT is an effective way to prevent recurrence. Shikowitz used PDT with intravenous photosensitizer dihematoporphyrin ether (DHE) to treat 48 patients with RRP and achieved encouraging efficacy [17]. However, the method was not widely used because of the damage of the skin by light. With the appearance of the second-generation photosensitizers like 5-ALA, which is for local use, and with fewer adverse reactions compared with the first generation, ALA-PDT has been widely used in recent years and there are also some reports proving its efficacy in preventing the recurrence of RRP in juveniles [18, 19]. It is reported that oxidative DNA damage does not play a significant role in photocarcinogenesis and photodynamic therapy with 5-ALA does not in itself increase the risk of malignant transformation [20]. In fact, PDT has been investigated for the past 30 years as an unconventional treatment for cancer [21, 22]. Therefore, we used PDT to eliminate potential malignant cells and prevent canceration in our case. The 15-month follow-up showed that there was no recurrence in our patient, which satisfied our expectations.

Pain is the most reported side effect of PDT. Pain during illumination can be significant and can interfere with desire for and completion of PDT treatments [23]. The feeling of discomfort most likely occurs during irradiation and was described as a prickling, burning, initial stinging, sharp, and painful sensation [24, 25]. In Waters' study [26], 16% of patients suffered severe pain during topical PDT treatments and an additional 50% of patients described treatment as moderately painful on a semiquantitative scale. At the last phase, the pain was usually replaced by a throbbing sensation persisting for several hours [27]. All the side effects and painful experience lower the evaluation of PDT. However, in our case, PDT was given under general anesthesia, which did not cause any trouble for our patient. Therefore, in RRP, the recommendation of topical PDT treatment under general anesthesia may be a good way to reduce patients' bitter experience.

Though PDT has been proved to be an effective way to prevent the recurrence of RRP in many reported cases, in most of the literature, the number of cases was small, the follow-up time was not enough, and the inclusion criteria, treatment strategies, photosensitizer, and evaluation method of curative effect varied in different references, thus limiting its accurate conclusion whether PDT can effectively treat RRP and prevent the recurrence in long term. The same weakness in our case needs to be mentioned. Though the follow-up period was about 15 months, the follow-up time is too short to be a single case to prove the patient had been cured without recurrence and canceration. Further research including larger samples is required to prove the long-term effectiveness of this treatment.

In conclusion, RRP in adult should be distinguished from laryngeal malignant tumors, especially when high-risk HPV types are found in the lesion. After therapy, a long-term follow-up is necessary to prevent the recurrence and risk of malignant transformation. Also, PDT might be an effective way in preventing the recurrence of RRP.
